# Supplementary material for: Irritable Bowel Syndrome and the Gut Microbiome: A Comprehensive Review
Source: J Clin Med. 2023 Mar 28;12(7):2558. doi: 10.3390/jcm12072558 (PMC10095554; doi:10.3390/jcm12072558)
Supplement: Supplementary file 1 [file jcm-12-02558-s001.zip › Supplemental File_ClinicalTrials_IBS and FMT compilation accessed 03092023.pdf]

ClinicalTrials.gov Search Results 03/09/2023

1	NCT02092402	Fecal Microbiota Transplantation in Patients With Irritable Bowel Syndrome<br>Study Documents:	Title Acronym:<br>Other Ids:<br>2013/180	Completed	•Irritable Bowel Syndrome (IBS)	•Other: Fecal transplantation	Study Type:<br>Interventional<br>Phase:<br>Not Applicable<br>Study Design:<br>•Allocation: Randomized<br>•Intervention Model: Parallel Assignment<br>•Masking: Quadruple (Participant, Care Provider, Investigator, Outcomes Assessor)<br>•Primary Purpose: Treatment<br>Outcome Measures:<br>•Effect of fecal microbiota transplantation on the symptoms of IBS patients using the IBS version of the gastrointestinal symptom rating scale (GSRS-IBS)<br>•Effect of fecal transplantation on IBS patients' visceral perception (Pain scores on the visual analogue scale during barostat procedure)<br>•Effect of fecal transplantation on IBS patients' composition of mucosal microbiota (HITChip analysis)<br>•Effect of fecal transplantation on IBS patients' composition of fecal microbiota (HITChip analysis)<br>•Effect of fecal transplantation on IBS patients' mucosal immune cell composition (lymphocyte fingerprinting using flow cytometry)<br>•Effect of fecal microbiota transplantation on the symptoms of IBS patients using the IBS - severity scoring system (IBS-SSS)<br>•Effect of fecal microbiota transplantation on the symptoms of IBS patients using the health-related quality of life questionnaire for IBS patients (IBS-QOL)<br>•Effect of fecal microbiota transplantation on the symptoms of IBS patients using the hospital and	Enrollment:<br>17<br>Age:<br>18 Years to 65 Years (Adult, Older Adult)<br>Sex:<br>All	•Örebro University, Sweden	•Other	Study Start:<br>September 2013<br>Primary Completion:<br>November 2016<br>Study Completion:<br>June 2019<br>First Posted:<br>March 20, 2014<br>Results First Posted:<br>No Results Posted<br>Last Update Posted:<br>August 28, 2019	•University Hospital Örebro, Örebro, Sweden

	NCT Number	Title	Other Names	Status	Conditions	Interventions	Characteristics	Population	Sponsor/ Collaborators	Funder Type	Dates	Locations
2	NCT03074227	The FAIS-Trial: Faecal Microbiota Transplantation (FMT) in Adolescents With Refractory Irritable Bowel Syndrome (IBS)<br><br>Study Documents:	Title Acronym: FAIS<br><br>Other Ids: FAIS2016	Recruiting	• Irritable Bowel Syndrome	• Other: Allogeneic faecal transplantation<br>• Other: Autologous faecal transplantation	Study Type: Interventional<br><br>Phase: Not Applicable<br><br>Study Design:<br>• Allocation: Randomized<br>• Intervention Model: Parallel Assignment<br>• Masking: Quadruple (Participant, Care Provider, Investigator, Outcomes Assessor)<br>• Primary Purpose: Treatment<br><br>Outcome Measures:<br>• The proportion of patients with > 50% reduction of their abdominal pain intensity and pain frequency at t=12 weeks after the first faecal transplantation<br>• Intra-individual changes in faecal gut microbiota composition<br>• Adverse events<br>• The proportion of patients with > 50% reduction of their abdominal pain intensity and pain frequency<br>• Total IBS-SSS score<br>• Health related quality of life<br>• Depression and anxiety<br>• Absence of school or work, health care resources and costs<br>• Adequate relief<br>• Number of participants with treatment-related adverse events as assessed by CRP, liver profile and renal profile	Enrollment: 30<br><br>Age: 16 Years to 21 Years (Child, Adult)<br><br>Sex: All	• Academisch Medisch Centrum - Universiteit van Amsterdam (AMC-UvA)	• Other	Study Start: November 23, 2017<br><br>Primary Completion: August 31, 2022<br><br>Study Completion: August 31, 2022<br><br>First Posted: March 8, 2017<br><br>Results First Posted: No Results Posted<br><br>Last Update Posted: February 22, 2022	• AMC, Amsterdam, Noord Holland, Netherlands

	NCT Number	Title	Other Names	Status	Conditions	Interventions	Characteristics	Population	Sponsor/ Collaborators	Funder Type	Dates	Locations
3	NCT02299973	Fecal Microbiota Transplantation in Irritable Bowel Syndrome With Bloating<br><br>Study Documents:	Title Acronym:<br><br>Other Ids:<br>UGent_Gastro_001	Completed	•Irritable Bowel Syndrome	•Procedure: FMT with donor stool<br>•Procedure: FMT with own stool	Study Type:<br>Interventional<br><br>Phase:<br>Not Applicable<br><br>Study Design:<br>•Allocation: Randomized<br>•Intervention Model: Parallel Assignment<br>•Masking: Quadruple (Participant, Care Provider, Investigator, Outcomes Assessor)<br>•Primary Purpose: Treatment<br><br>Outcome Measures:<br>•Reduction of overall IBS symptoms (Key question 1)<br>•Reduction of abdominal bloating (Key question 2)<br>•Changes in fecal microbiome composition (Illumina sequencing)<br>•Changes in IBS symptom scores at three months after FMT<br>•Changes in IBS symptom scores at six months post FMT<br>•Changes in IBS symtom scores at 9 months post FMT<br>•Changes in IBS symptom scores at 1 year post FMT<br>•Composition of mucosal-adherent microbiota (Illumina sequencing)<br>•Changes of IBS symptom scores in patients who undergo an off-trial FMT	Enrollment:<br>64<br><br>Age:<br>18 Years to 75 Years (Adult, Older Adult)<br><br>Sex:<br>All	•University Hospital, Ghent	•Other	Study Start:<br>October 2014<br><br>Primary Completion:<br>December 2017<br><br>Study Completion:<br>December 2017<br><br>First Posted:<br>November 24, 2014<br><br>Results First Posted:<br>No Results Posted<br><br>Last Update Posted:<br>December 8, 2017	•Ghent University Hospital, Ghent, Belgium

	NCT Number	Title	Other Names	Status	Conditions	Interventions	Characteristics	Population	Sponsor/ Collaborators	Funder Type	Dates	Locations
4	NCT02328547	Fecal Microbiota Transplantation for the Treatment of Diarrhea-Predominant Irritable Bowel Syndrome<br><br>Study Documents:<br>• Study Protocol and Statistical Analysis Plan	Title Acronym:<br><br>Other Ids:<br>2014-3941	Completed	• Irritable Bowel Syndrome	• Drug: Fecal microbiota transplantation capsules<br>• Drug: Placebo capsules	Study Type:<br>Interventional<br><br>Phase:<br>Phase 2<br><br>Study Design:<br>• Allocation: Randomized<br>• Intervention Model: Crossover Assignment<br>• Masking: Quadruple (Participant, Care Provider, Investigator, Outcomes Assessor)<br>• Primary Purpose: Treatment<br><br>Outcome Measures:<br>• Within and Between Group Comparisons of Disease Severity as Determined by Irritable Bowel Syndrome-Symptom Severity Score (IBS-SSS)<br>• Within and Between Group Comparisons of Quality of Life as Determined by the Irritable Bowel Syndrome-Quality of Life (IBS-QOL) Score<br>• Intestinal Microbiota Composition Pre- and Post-FMT (Fecal Microbiota Transplantation)<br>• Anxiety as Measured by the Hospital Anxiety and Depression Scale (HADS). HADS-A (Anxiety)<br>• Depression as Measured by the Hospital Anxiety and Depression Scale (HADS). HADS-D (Depression)<br>• Bowel Consistency as Measured by the Bristol Stool Form Scale (BSFS)<br>• Number of Participants With Adverse Events as a Measure of Safety and Tolerability<br>• Satisfaction With Fecal Microbiota Transplantation (FMT)<br>• Change in Bowel Habits and Abdominal Pain After Fecal Microbiota Transplantation (FMT)<br>• Number of Doctor or Emergency Department (ED) Visits Post-Fecal Microbiota Transplantation (Post-FMT) for Irritable	Enrollment:<br>48<br><br>Age:<br>19 Years to 65 Years (Adult, Older Adult)<br><br>Sex:<br>All	• Montefiore Medical Center	• Other	Study Start:<br>May 2015<br><br>Primary Completion:<br>October 2017<br><br>Study Completion:<br>March 2018<br><br>First Posted:<br>December 31, 2014<br><br>Results First Posted:<br>July 2, 2019<br><br>Last Update Posted:<br>July 2, 2019	• Medical Research Center of Connecticut, Hamden, Connecticut, United States<br>• Montefiore Medical Center, Bronx, New York, United States<br>• Concorde Medical Group, New York, New York, United States

	NCT Number	Title	Other Names	Status	Conditions	Interventions	Characteristics	Population	Sponsor/ Collaborators	Funder Type	Dates	Locations
5	NCT04691544	Donor Versus Autologous Fecal Microbiota Transplantation for Irritable Bowel Syndrome<br>Study Documents:	Title Acronym:<br>Other Ids:<br>183984	Active, not recruiting	• Irritable Bowel Syndrome	• Biological: Fecal microbiota transplantation (FMT)	Study Type:<br>Interventional<br>Phase:<br>Phase 3<br>Study Design:<br>• Allocation: Randomized<br>• Intervention Model: Parallel Assignment<br>• Masking: Triple (Participant, Care Provider, Investigator)<br>• Primary Purpose: Treatment<br>Outcome Measures:<br>• Proportion in the donorFMT (dFMT) versus autologousFMT (aFMT) group with #75 points decrease in the Irritable Bowel Syndrome Symptom Severity Scale (IBS-SSS) day 90 after treatment when compared to the score 8 days before treatment<br>• Proportion in dFMT versus aFMT group with #75 points decrease in the Irritable Bowel Syndrome Symptom Severity Scale (IBS-SSS) day 365 after treatment when compared to the score 8 days before treatment<br>• Proportion of patients in dFMT versus aFMT group with a #14 points increase in the IBS-Qualiy of Life (IBS-QoL) day 90 after treatment when compared to the score 8 days before treatment<br>• Proportion in the dFMT vs aFMT group with 2 or more weeks with treatment success in Adequate relief by the Global Improvement Scale and Abdominal pain day 69, 76, 83 and 90 after treatment. For treatment success criteria A. and B. have to be fulfilled<br>• Proportion of adverse events and serious adverse events in the dFMT versus aFMT group from treatment and until day 90 after treatment<br>• Change in the dFMT vs aFMT group in mean stool	Enrollment:<br>450<br>Age:<br>18 Years to 65 Years (Adult, Older Adult)<br>Sex:<br>All	• University Hospital of North Norway<br>• Oslo University Hospital<br>• Sorlandet Hospital HF<br>• Haukeland University Hospital<br>• Alesund Hospital	• Other	Study Start:<br>May 5, 2021<br>Primary Completion:<br>August 1, 2023<br>Study Completion:<br>December 31, 2026<br>First Posted:<br>December 31, 2020<br>Results First Posted:<br>No Results Posted<br>Last Update Posted:<br>October 28, 2022	• Ålesund Hospital, Ålesund, Norway

	NCT Number	Title	Other Names	Status	Conditions	Interventions	Characteristics	Population	Sponsor/ Collaborators	Funder Type	Dates	Locations
6	NCT03613545	Fecal Microbiota Transplantation for Irritable Bowel Syndrome<br><br>Study Documents:	Title Acronym:<br><br>Other Ids:<br>K-2017-078-02	Recruiting	• Irritable Bowel Syndrome<br>• Fecal Microbiota Transplantation	• Procedure: fecal microbiota transplantation<br>• Procedure: Infusion of sham<br>• Drug: probiotics, antibiotics or antidepressants	Study Type:<br>Interventional<br><br>Phase:<br>• Phase 2<br>• Phase 3<br><br>Study Design:<br>• Allocation: Randomized<br>• Intervention Model: Parallel Assignment<br>• Masking: Single (Participant)<br>• Primary Purpose: Treatment<br><br>Outcome Measures:<br>• Change in Irritable Bowel Syndrome assessed by Symptom Severity Score (IBS-SSS)<br>• Change in Irritable Bowel Syndrome assessed by Quality of Life (IBS-QOL) Questionnaire Scores<br>• Change in Depression and Anxiety assessed by Hamilton Depression Rating Scale (HAMD) and Hamilton Anxiety Rating Scale(HAMA)	Enrollment:<br>120<br><br>Age:<br>18 Years to 75 Years (Adult, Older Adult)<br><br>Sex:<br>All	• Guangzhou First People's Hospital	• Other	Study Start:<br>August 10, 2018<br><br>Primary Completion:<br>December 31, 2028<br><br>Study Completion:<br>December 31, 2030<br><br>First Posted:<br>August 3, 2018<br><br>Results First Posted:<br>No Results Posted<br><br>Last Update Posted:<br>August 3, 2021	• Guangzhou First People's Hospital, Guangzhou, Guangdong, China

	NCT Number	Title	Other Names	Status	Conditions	Interventions	Characteristics	Population	Sponsor/ Collaborators	Funder Type	Dates	Locations
7	NCT02423421	Faecal Microbiota Transplantation in Irritable Bowel Syndrome<br><br>Study Documents:	Title Acronym:<br><br>Other Ids:<br>APC053	Unknown status	• Irritable Bowel Syndrome	• Other: Faecal microbiota transplantation	Study Type:<br>Interventional<br><br>Phase:<br>Phase 2<br><br>Study Design:<br>• Allocation: Randomized<br>• Intervention Model: Parallel Assignment<br>• Masking: Double (Participant, Outcomes Assessor)<br>• Primary Purpose: Treatment<br><br>Outcome Measures:<br>• Global Assessment of relief of IBS symptoms.<br>• Primary symptoms of IBS<br>• Quality of life<br>• Depression and Anxiety<br>• Safety as measured by occurrence of adverse events	Enrollment:<br>50<br><br>Age:<br>18 Years to 65 Years (Adult, Older Adult)<br><br>Sex:<br>All	• University College Cork	• Other	Study Start:<br>March 2015<br><br>Primary Completion:<br>June 2016<br><br>Study Completion:<br>June 2016<br><br>First Posted:<br>April 22, 2015<br><br>Results First Posted:<br>No Results Posted<br><br>Last Update Posted:<br>April 22, 2015	• Alimentary Pharmabiotic Centre, University College Cork, Cork, Ireland
8	NCT02847481	A Study to Evaluate Fecal Microbiota Transplantation Engraftment in IBS<br><br>Study Documents:	Title Acronym:<br><br>Other Ids:<br>2015P000211	Completed	• Irritable Bowel Syndrome	• Procedure: fecal microbiota transplantation<br>• Drug: placebo fecal microbiota transplantation<br>• Procedure: FMT with antibiotic pre-treatment (v1)<br>• Procedure: FMT with antibiotic pre-treatment (v2)	Study Type:<br>Interventional<br><br>Phase:<br>Phase 1<br><br>Study Design:<br>• Allocation: Randomized<br>• Intervention Model: Parallel Assignment<br>• Masking: Double (Participant, Investigator)<br>• Primary Purpose: Treatment<br><br>Outcome Measures:<br>stable engraftment of donor microbiota	Enrollment:<br>80<br><br>Age:<br>18 Years and older (Adult, Older Adult)<br><br>Sex:<br>All	• Beth Israel Deaconess Medical Center<br>• OpenBiome	• Other<br>• Industry	Study Start:<br>May 2016<br><br>Primary Completion:<br>August 5, 2018<br><br>Study Completion:<br>August 5, 2018<br><br>First Posted:<br>July 28, 2016<br><br>Results First Posted:<br>No Results Posted<br><br>Last Update Posted:<br>December 23, 2019	• Beth Israel Deaconess Medical Center, Boston, Massachusetts, United States

	NCT Number	Title	Other Names	Status	Conditions	Interventions	Characteristics	Population	Sponsor/ Collaborators	Funder Type	Dates	Locations
9	NCT02154867	Fecal Microbial Transplantation in Treatment of Irritable Bowel Syndrome; a Double Blinded Placebo Controlled Trial.<br><br>Study Documents:	Title Acronym: REFIT<br><br>Other Ids: 2013/971/REK	Completed	• Irritable Bowel Syndrome	• Biological: Fecal transplantation<br>• Other: Placebo fecal transplant	Study Type: Interventional<br><br>Phase: Phase 2<br><br>Study Design:<br>• Allocation: Randomized<br>• Intervention Model: Parallel Assignment<br>• Masking: Quadruple (Participant, Care Provider, Investigator, Outcomes Assessor)<br>• Primary Purpose: Treatment<br><br>Outcome Measures:<br>• Change in subjective symptom score<br>• Microbiome profile change<br>• Long term effects of fecal transplantation<br>• Safety of fecal transplantation in IBS	Enrollment: 90<br><br>Age: 18 Years to 75 Years (Adult, Older Adult)<br><br>Sex: All	• University Hospital of North Norway	• Other	Study Start: December 2014<br><br>Primary Completion: October 2016<br><br>Study Completion: December 2016<br><br>First Posted: June 3, 2014<br><br>Results First Posted: No Results Posted<br><br>Last Update Posted: January 13, 2017	• University Hospital of North Norway, Harstad, Norway
10	NCT02651740	Gut Microbiota Reconstruction in the Treatment of Irritable Bowel Syndrome With Predominant Diarrhea<br><br>Study Documents:	Title Acronym:<br><br>Other Ids: SXZ-WSD01-2015	Recruiting	• Irritable Bowel Syndrome	• Drug: Rifaximin<br>• Procedure: Fecal microbiota transplantation	Study Type: Interventional<br><br>Phase:<br>• Phase 2<br>• Phase 3<br><br>Study Design:<br>• Allocation: N/A<br>• Intervention Model: Single Group Assignment<br>• Masking: None (Open Label)<br>• Primary Purpose: Treatment<br><br>Outcome Measures:<br>• Number of patients with relief of IBS condition<br>• Number of patients with relief of IBS related anxiety or depression status<br>• Number of patients with relief of IBS single symptoms	Enrollment: 10<br><br>Age: 18 Years to 65 Years (Adult, Older Adult)<br><br>Sex: All	• Shanghai Zhongshan Hospital	• Other	Study Start: April 2016<br><br>Primary Completion: July 2023<br><br>Study Completion: December 2023<br><br>First Posted: January 11, 2016<br><br>Results First Posted: No Results Posted<br><br>Last Update Posted: December 30, 2021	• Zhongshan Hospital, Fudan University, Shanghai, China

	NCT Number	Title	Other Names	Status	Conditions	Interventions	Characteristics	Population	Sponsor/ Collaborators	Funder Type	Dates	Locations
11	NCT02788071	Effect of Fecal Microbiota Transplantation in Irritable Bowel Syndrome<br><br>Study Documents:	Title Acronym:<br><br>Other Ids:<br>H-15016343	Completed	• Irritable Bowel Syndrome	• Dietary Supplement: FMT capsules<br>• Dietary Supplement: FMT placebo	Study Type:<br>Interventional<br><br>Phase:<br>• Phase 2<br>• Phase 3<br><br>Study Design:<br>• Allocation: Randomized<br>• Intervention Model: Parallel Assignment<br>• Masking: Quadruple (Participant, Care Provider, Investigator, Outcomes Assessor)<br>• Primary Purpose: Treatment<br><br>Outcome Measures:<br>• symptoms score<br>• Change in microbiota diversity<br>• Microbiota diversity IBS patients<br>• Microbiota diversity in healthy donors<br>• Change in Irritable Bowel Syndrome-Quality of Life (IBS-QOL) Questionnaire Scores	Enrollment:<br>52<br><br>Age:<br>18 Years to 60 Years (Adult)<br><br>Sex:<br>All	• Aleris-Hamlet Hospitaler København<br>• Hvidovre University Hospital<br>• University of Aarhus	• Other	Study Start:<br>October 2016<br><br>Primary Completion:<br>July 2017<br><br>Study Completion:<br>July 2017<br><br>First Posted:<br>June 2, 2016<br><br>Results First Posted:<br>No Results Posted<br><br>Last Update Posted:<br>August 2, 2017	• Aleris Hamlet Hospitaler, København, Copenhagen, Søborg, Denmark

	NCT Number	Title	Other Names	Status	Conditions	Interventions	Characteristics	Population	Sponsor/ Collaborators	Funder Type	Dates	Locations
12	NCT05461833	FMT for Post-infectious IBS<br><br>Study Documents:	Title Acronym:<br><br>Other Ids:<br>FMT-PI-IBS	Completed	•Irritable Bowel Syndrome	•Biological: Fecal transplantation<br>•Drug: OTILONII BROMIDUM<br>•Dietary Supplement: multi-strain probiotic	Study Type:<br>Interventional<br><br>Phase:<br>Not Applicable<br><br>Study Design:<br>•Allocation: Randomized<br>•Intervention Model: Parallel Assignment<br>•Masking: None (Open Label)<br>•Primary Purpose: Treatment<br><br>Outcome Measures:<br>•Change in Irritable bowel syndrome severity scoring system (IBS-SSS)<br>•assesment of response rate<br>•Change in BS Quality of Life Scale (IBS-QoL)<br>•Change in Fatigue Assessment Scale (FAS)<br>•Bacteriology measured in the stool flora by specialized non-culture techniques<br>•Microbiome profile change	Enrollment:<br>59<br><br>Age:<br>18 Years to 65 Years (Adult, Older Adult)<br><br>Sex:<br>All	•Bogomolets National Medical University<br>•Ukrainian Research and Practical Centre of Endocrine Surgery, Transplantation of Endocrine Organs and Tissues of the Ministry of Health of Ukraine	•Other	Study Start:<br>September 1, 2020<br><br>Primary Completion:<br>December 31, 2021<br><br>Study Completion:<br>January 15, 2022<br><br>First Posted:<br>July 18, 2022<br><br>Results First Posted:<br>No Results Posted<br><br>Last Update Posted:<br>July 18, 2022	•Bogomolets National Medical University, Kyiv, Ukraine<br>•Ukrainian Research and Practical Centre of Endocrine Surgery, Transplantation of Endocrine Organs and Tissues of the Ministry of Health of Ukraine, Kyiv, Ukraine

	NCT Number	Title	Other Names	Status	Conditions	Interventions	Characteristics	Population	Sponsor/ Collaborators	Funder Type	Dates	Locations
13	NCT04890405	Clinical Study of Selective Fecal Microbiota Transplantation in the Treatment of Irritable Bowel Syndrome.<br>Study Documents:	Title Acronym:<br>Other Ids: TMMU-DP-GI-JZ-001	Not yet recruiting	• Irritable Bowel Syndrome Variant of Childhood	• Procedure: Standardized FMT<br>• Combination Product: Precision Flora Transplantation	Study Type: Interventional<br>Phase: Not Applicable<br>Study Design:<br>• Allocation: Randomized<br>• Intervention Model: Parallel Assignment<br>• Masking: Single (Outcomes Assessor)<br>• Primary Purpose: Treatment<br>Outcome Measures:<br>• IBS-SSS score<br>• Clinical remission rate<br>• GSRS score<br>• IBS-QoL score<br>• PHQ-9<br>• GAD-7<br>• SAS<br>• SDS<br>• Intestinal flora	Enrollment: 70<br>Age: 18 Years to 75 Years (Adult, Older Adult)<br>Sex: All	• Yanling Wei<br>• Third Military Medical University	• Other	Study Start: May 20, 2021<br>Primary Completion: October 1, 2021<br>Study Completion: October 1, 2022<br>First Posted: May 18, 2021<br>Results First Posted: No Results Posted<br>Last Update Posted: May 18, 2021	

	NCT Number	Title	Other Names	Status	Conditions	Interventions	Characteristics	Population	Sponsor/ Collaborators	Funder Type	Dates	Locations
14	NCT04899869	Faecal Microbiota Transplantation in Irritable Bowel Syndrome<br><br>Study Documents:	Title Acronym: MISCEAT<br><br>Other Ids: INT_TN_001	Recruiting	• Irritable Bowel Syndrome With Diarrhea<br>• Irritable Bowel Syndrome Mixed	• Other: Faecal microbiota transplantation with active study microbiota first<br>• Other: Faecal microbiota transplantation with inactive autoclaved study microbiota first<br>• Other: Faecal microbiota transplantation with inactive autoclaved study microbiota only	Study Type: Interventional<br><br>Phase: Not Applicable<br><br>Study Design:<br>• Allocation: Randomized<br>• Intervention Model: Crossover Assignment<br>• Masking: Triple (Participant, Care Provider, Investigator)<br>• Primary Purpose: Treatment<br><br>Outcome Measures:<br>• Change in the IBS severity symptom score (IBS-SSS)<br>• The acute change in the IBS severity symptom score (IBS-SSS)<br>• The long-term change in the IBS severity symptom score (IBS-SSS)<br>• Change in number of loose stools per day<br>• Change in stool consistency<br>• Change in abdominal pain<br>• Change in frequency of bloating per week<br>• Change in Body Mass Index<br>• Change in waist circumference<br>• Change in body fat mass estimated by skinfold thickness measuring<br>• and 5 more	Enrollment: 99<br><br>Age: 18 Years to 65 Years (Adult, Older Adult)<br><br>Sex: All	• Thomayer University Hospital<br>• Charles University, Czech Republic	• Other	Study Start: June 17, 2021<br><br>Primary Completion: June 30, 2023<br><br>Study Completion: December 31, 2023<br><br>First Posted: May 25, 2021<br><br>Results First Posted: No Results Posted<br><br>Last Update Posted: June 9, 2022	• Thomayer University Hospital, Prague, Czechia

	NCT Number	Title	Other Names	Status	Conditions	Interventions	Characteristics	Population	Sponsor/ Collaborators	Funder Type	Dates	Locations
15	NCT05361785	Fecal Microbiota Transplantation for Irritable Bowel Syndrome Associated Food Intolerance<br><br>Study Documents:	Title Acronym: FinFMT-IBS<br><br>Other Ids: 1480/2021	Recruiting	• Irritable Bowel Syndrome<br>• Fecal Microbiota Transplantation	• Biological: FMT	Study Type: Interventional<br><br>Phase: Not Applicable<br><br>Study Design:<br>• Allocation: Randomized<br>• Intervention Model: Single Group Assignment<br>• Masking: Single (Investigator)<br>• Primary Purpose: Treatment<br><br>Outcome Measures:<br>• The effect of FMT for tolerance of FODMAPs in the IBS patients' diet<br>• Microbial components explaining the successful broadening of FODMAP diet in IBS patients.<br>• GI symptoms and bacterial fermentaiton status in IBS	Enrollment: 45<br><br>Age: 18 Years to 75 Years (Adult, Older Adult)<br><br>Sex: All	• Helsinki University Central Hospital<br>• Turku University Hospital<br>• University of Helsinki<br>• Paijat-Hame Hospital District	• Other	Study Start: April 30, 2022<br><br>Primary Completion: July 31, 2024<br><br>Study Completion: July 31, 2026<br><br>First Posted: May 5, 2022<br><br>Results First Posted: No Results Posted<br><br>Last Update Posted: May 13, 2022	• Helsinki University Hospital, Helsinki, Helsinki And Uusimaa, Finland

	NCT Number	Title	Other Names	Status	Conditions	Interventions	Characteristics	Population	Sponsor/ Collaborators	Funder Type	Dates	Locations
16	NCT03125564	FMT for Patients With IBS With Fecal and Mucosal Microbiota Assessment<br>Study Documents:	Title Acronym:<br>Other Ids: FMT-IBS study	Active, not recruiting	• Irritable Bowel Syndrome<br>• Fecal Microbiota Transplantation	• Procedure: Fecal Microbiota Transplantation<br>• Procedure: Sham<br>• Procedure: Fecal and Mucosal Microbiota Assessment	Study Type: Interventional<br>Phase: Not Applicable<br>Study Design:<br>• Allocation: Randomized<br>• Intervention Model: Parallel Assignment<br>• Masking: Double (Participant, Investigator)<br>• Primary Purpose: Treatment<br>Outcome Measures:<br>• the proportion of responders<br>• The proportion of patients who had adequate relief of general IBS symptoms<br>• Assess the onset and duration of relief of general IBS symptoms<br>• The proportion of patients who had improvement on abdominal bloating<br>• Assess the onset and duration of abdominal bloating relief<br>• Assess the Abdominal pain between two groups<br>• Assess the Stool consistency between two groups<br>• Health-related quality of life in patients with irritable bowel syndrome<br>• Assess the level of anxiety between two groups<br>• Assess the change of abdominal pain scores in patients who undergo open-label FMT<br>• and 6 more	Enrollment: 56<br>Age: 18 Years and older (Adult, Older Adult)<br>Sex: All	• Chinese University of Hong Kong	• Other	Study Start: April 12, 2017<br>Primary Completion: September 16, 2022<br>Study Completion: March 16, 2023<br>First Posted: April 24, 2017<br>Results First Posted: No Results Posted<br>Last Update Posted: October 17, 2022	• The Chinese University of Hong Kong, Sha Tin, Hong Kong

	NCT Number	Title	Other Names	Status	Conditions	Interventions	Characteristics	Population	Sponsor/ Collaborators	Funder Type	Dates	Locations
17	NCT03333291	Fecal Transplantation in Patients With IBS<br>Study Documents:	Title Acronym:<br>Other Ids: 2013/1497	Completed	•IBS - Irritable Bowel Syndrome	•Dietary Supplement: fecal suspension	Study Type: Interventional<br>Phase: Not Applicable<br>Study Design:<br>•Allocation: N/A<br>•Intervention Model: Single Group Assignment<br>•Masking: None (Open Label)<br>•Primary Purpose: Treatment<br>Outcome Measures:<br>•Stool microbiota changes<br>•Global improvement in IBS symptoms	Enrollment: 14<br>Age: 18 Years to 70 Years (Adult, Older Adult)<br>Sex: All	•Haukeland University Hospital<br>•Helse Vest	•Other	Study Start: October 1, 2016<br>Primary Completion: June 15, 2017<br>Study Completion: June 30, 2017<br>First Posted: November 6, 2017<br>Results First Posted: No Results Posted<br>Last Update Posted: November 6, 2017	•Helse Bergen HF, Haukeland University Hospital, Bergen, Norway
18	NCT05088434	Fecal Microbiota Transplantation and ACHIM for Manipulating Gut Microbiota in IBS Patients<br>Study Documents:	Title Acronym:<br>Other Ids: 2016/1914	Completed	•Irritable Bowel Syndrome<br>•Dysbiosis	•Dietary Supplement: Anaerobically Cultivated Human Intestinal Microbiota (ACHIM)<br>•Dietary Supplement: Donor fecal microbiota transplantation<br>•Dietary Supplement: Placebo	Study Type: Interventional<br>Phase: Not Applicable<br>Study Design:<br>•Allocation: Randomized<br>•Intervention Model: Parallel Assignment<br>•Masking: Triple (Participant, Care Provider, Investigator)<br>•Primary Purpose: Treatment<br>Outcome Measures:<br>•Gut microbiota analysis<br>•Irritable bowel syndrome Symptom Severity Scale (IBS-SSS)<br>•Bristol stool form scale<br>•Short form of Nepean Dyspepsia Index (SF-NDI)<br>•Eysenck Personality Questionnaire Neuroticism (EPQ-N-12_<br>•Hospital Anxiety and Depression Scale (HADS)	Enrollment: 62<br>Age: 18 Years to 65 Years (Adult, Older Adult)<br>Sex: All	•Haukeland University Hospital	•Other	Study Start: January 1, 2017<br>Primary Completion: August 30, 2020<br>Study Completion: June 30, 2021<br>First Posted: October 22, 2021<br>Results First Posted: No Results Posted<br>Last Update Posted: October 22, 2021	•Haukeland Unversity Hospital, Bergen, Norway

	NCT Number	Title	Other Names	Status	Conditions	Interventions	Characteristics	Population	Sponsor/ Collaborators	Funder Type	Dates	Locations
19	NCT04236843	Faecal Microbiota Transplantation (FMT) in Patients With IBSmechanism(s) of Action<br>Study Documents:	Title Acronym:<br>Other Ids: Helse Fonna	Completed	• Irritable Bowel Syndrome	• Dietary Supplement: Feces	Study Type: Interventional<br>Phase: Not Applicable<br>Study Design:<br>• Allocation: Randomized<br>• Intervention Model: Parallel Assignment<br>• Masking: None (Open Label)<br>• Primary Purpose: Treatment<br>Outcome Measures:<br>• Change in IBS-SSS total score<br>• Change in the Dysbiosis index	Enrollment: 186<br>Age: 18 Years to 75 Years (Adult, Older Adult)<br>Sex: All	• Helse Fonna<br>• University of Bergen	• Other	Study Start: February 3, 2020<br>Primary Completion: March 25, 2022<br>Study Completion: March 25, 2022<br>First Posted: January 22, 2020<br>Results First Posted: No Results Posted<br>Last Update Posted: November 14, 2022	• Helse Fonna, Haugesund, Norway
20	NCT03561519	FMT in the Treatment of IBS<br>Study Documents:	Title Acronym: FMT-IBS<br>Other Ids: 40/13/03/01/15	Completed	• Irritable Bowel Syndrome	• Other: Fecal Microbiota Transplantation (FMT)<br>• Other: Placebo	Study Type: Interventional<br>Phase: Not Applicable<br>Study Design:<br>• Allocation: Randomized<br>• Intervention Model: Parallel Assignment<br>• Masking: Quadruple (Participant, Care Provider, Investigator, Outcomes Assessor)<br>• Primary Purpose: Treatment<br>Outcome Measures: IBS symptom relieve	Enrollment: 52<br>Age: 18 Years to 75 Years (Adult, Older Adult)<br>Sex: All	• Joint Authority for Päijät-Häme Social and Health Care<br>• Helsinki University Central Hospital<br>• University of Helsinki	• Other	Study Start: August 27, 2015<br>Primary Completion: July 1, 2018<br>Study Completion: July 1, 2018<br>First Posted: June 19, 2018<br>Results First Posted: No Results Posted<br>Last Update Posted: August 15, 2018	• Helsinki University Hospital, Helsinki, Finland

	NCT Number	Title	Other Names	Status	Conditions	Interventions	Characteristics	Population	Sponsor/ Collaborators	Funder Type	Dates	Locations
21	NCT05740319	Efficacy and Safety Evaluation of Fecal Microbiota Transplantation in Irritable Bowel Syndrome<br><br>Study Documents:	Title Acronym:<br><br>Other Ids:<br>B2022-507R	Recruiting	•Irritable Bowel Syndrome	•Other: FMT	Study Type:<br>Interventional<br><br>Phase:<br>Not Applicable<br><br>Study Design:<br>•Allocation: N/A<br>•Intervention Model: Single Group Assignment<br>•Masking: None (Open Label)<br>•Primary Purpose: Treatment<br><br>Outcome Measures:<br>•Change from baseline IBS symptom severity scale (IBS-SSS) score at 9 weeks<br>•Response rate at 9 weeks<br>•Change from baseline IBS symptom severity scale (IBS-SSS) score at 1 week, 1 month and 6 months<br>•Change from baseline Gastrointestinal Symptom Rating Scale (GSRS) score at 1 week, 1 month, 9 weeks and 6 months<br>•Change from baseline IBS-Quality of Life (IBS-QoL) score at 1 month, 3 months and 6 months<br>•Change from baseline Self-rating Anxiety Scale (SAS) score at 1 month, 3 months and 6 months<br>•Change from baseline Self-rating Depression Scale (SDS) score at 1 month, 3 months and 6 months<br>•Response rate at 1 week, 1month, 3 months and 6 months<br>•Change from baseline fecal microbiota composition at 1 week, 1 month and 6 months<br>•Change from baseline fecal metabolites at 1 week, 1 month and 6 months	Enrollment:<br>102<br><br>Age:<br>18 Years to 70 Years (Adult, Older Adult)<br><br>Sex:<br>All	•Shanghai Zhongshan Hospital	•Other	Study Start:<br>March 10, 2023<br><br>Primary Completion:<br>December 31, 2024<br><br>Study Completion:<br>December 31, 2024<br><br>First Posted:<br>February 23, 2023<br><br>Results First Posted:<br>No Results Posted<br><br>Last Update Posted:<br>February 23, 2023	•Zhongshan Hospital, Fudan University, Shanghai, Shanghai, China

	NCT Number	Title	Other Names	Status	Conditions	Interventions	Characteristics	Population	Sponsor/ Collaborators	Funder Type	Dates	Locations
22	NCT05174273	Neurocognitive Effects of FMT in MDD Patients With and Without IBS<br>Study Documents:	Title Acronym:<br>Other Ids: IMA-FMT-MDD/ IBS-2020	Recruiting	• Major Depressive Disorder<br>• Irritable Bowel Syndrome	• Biological: Fecal Microbiota Transplantation	Study Type: Interventional<br>Phase: • Phase 2 • Phase 3<br>Study Design: • Allocation: Non-Randomized • Intervention Model: Parallel Assignment • Masking: None (Open Label) • Primary Purpose: Other<br>Outcome Measures: • The Montgomery-Åsberg Depression Rating Scale (MADRS) • IBS Symptom Severity Scale (IBS-SSS) • Toronto Side Effect Scale (TSES) • IBS specific Quality of Life (IBS-QoL) • nuclear magnetic resonance (NMR) spectrometry	Enrollment: 180<br>Age: 18 Years to 60 Years (Adult)<br>Sex: All	• Valerie Taylor<br>• University of Calgary	• Other	Study Start: April 6, 2022<br>Primary Completion: December 2023<br>Study Completion: April 2024<br>First Posted: December 30, 2021<br>Results First Posted: No Results Posted<br>Last Update Posted: May 25, 2022	• University of Calgary, TRW building, Calgary, Alberta, Canada

	NCT Number	Title	Other Names	Status	Conditions	Interventions	Characteristics	Population	Sponsor/ Collaborators	Funder Type	Dates	Locations
23	NCT03822299	Effects of Faecal Microbiota Transplantation in Patients With IBS<br><br>Study Documents:	Title Acronym:<br><br>Other Ids: HelseFonna	Completed	• Irritable Bowel Syndrome	• Dietary Supplement: healthy feces microbiota	Study Type: Interventional<br><br>Phase: Not Applicable<br><br>Study Design:<br>• Allocation: Randomized<br>• Intervention Model: Parallel Assignment<br>• Masking: Single (Outcomes Assessor)<br>• Primary Purpose: Treatment<br><br>Outcome Measures:<br>• Global improvement in IBS symptoms as assessed by IBS Symptom Severity Scale (IBS-SSS)<br>• Global improvement in IBS symptoms as assessed by Birmingham Symptom scale questionnaire<br>• Quality of life as assessed by IBS quality of life (IBSQoL) questionnaire<br>• Quality of life as assessed by Short form of Nepean Dyspepsia Index (SF-NDI) questionnaires<br>• Fatigue as assessed by: Fatigue Assessment Scale (FAS) questionnaire<br>• Stool microbiota changes as assessed by the Dysbiosis index (DI)	Enrollment: 164<br><br>Age: 18 Years to 85 Years (Adult, Older Adult)<br><br>Sex: All	• Helse Fonna<br>• Helse Vest	• Other	Study Start: January 1, 2018<br><br>Primary Completion: April 30, 2019<br><br>Study Completion: May 5, 2019<br><br>First Posted: January 30, 2019<br><br>Results First Posted: No Results Posted<br><br>Last Update Posted: May 7, 2019	• Helse Fonna, Haugesund, Norway

	NCT Number	Title	Other Names	Status	Conditions	Interventions	Characteristics	Population	Sponsor/ Collaborators	Funder Type	Dates	Locations
24	NCT04011943	Fecal Microbiota Transplantation for Health Improvement (TFM3)<br>Study Documents:	Title Acronym: TFM3<br>Other Ids: TFM3	Unknown status	•Ulcerative Colitis<br>•Irritable Bowel Syndrome<br>•Crohn Disease<br>•Irritable Bowel	•Other: Fecal Microbiota Transplantation	Study Type: Interventional<br>Phase: Not Applicable<br>Study Design:<br>•Allocation: Non-Randomized<br>•Intervention Model: Parallel Assignment<br>•Masking: Single (Participant)<br>•Primary Purpose: Treatment<br>Outcome Measures:<br>•Ulcerative Colitis remission<br>•Crohn Disease remission<br>•Improvement in Ulcerative Colitis symptoms.<br>•Change in gut microbiome	Enrollment: 50<br>Age: 18 Years to 75 Years (Adult, Older Adult)<br>Sex: All	•Federal Research and Clinical Center of Physical-Chemical Medicine	•Other	Study Start: May 21, 2018<br>Primary Completion: December 1, 2020<br>Study Completion: December 1, 2020<br>First Posted: July 9, 2019<br>Results First Posted: No Results Posted<br>Last Update Posted: July 9, 2019	•FRCC PCM, Moscow, Russian Federation
25	NCT04014413	Safety and Efficacy of Fecal Microbiota Transplantation<br>Study Documents:	Title Acronym:<br>Other Ids: FMT-Pilot	Recruiting	•Crohn Disease<br>•Ulcerative Colitis<br>•Celiac Disease<br>•Irritable Bowel Syndrome<br>•Functional Dysphonia<br>•Constipation<br>•Clostridium Difficile Infection<br>•Diabetes Mellitus<br>•Obesity<br>•Multidrug - Resistant Infection<br>•and 17 more	•Procedure: Fecal Microbiota Transplantation	Study Type: Interventional<br>Phase: Not Applicable<br>Study Design:<br>•Allocation: Non-Randomized<br>•Intervention Model: Parallel Assignment<br>•Masking: None (Open Label)<br>•Primary Purpose: Treatment<br>Outcome Measures:<br>•The efficacy of FMT in treating dysbiosis-associated disorder will be assessed by number of patients who have improvement in clinical symptoms (depends on each disease as stated in outcome)<br>•Number of Participants With Treatment-Related Adverse Events as Assessed by CTCAE v4.0	Enrollment: 450<br>Age: Child, Adult, Older Adult<br>Sex: All	•Chinese University of Hong Kong	•Other	Study Start: July 15, 2019<br>Primary Completion: October 31, 2023<br>Study Completion: October 31, 2024<br>First Posted: July 10, 2019<br>Results First Posted: No Results Posted<br>Last Update Posted: February 14, 2022	•The Chinese University of Hong Kong, Hong Kong, Shatin, Hong Kong

	NCT Number	Title	Other Names	Status	Conditions	Interventions	Characteristics	Population	Sponsor/ Collaborators	Funder Type	Dates	Locations
26	NCT03763175	Efficacy and Safety of SYN-010 in IBS-C<br><br>Study Documents:<br>• Study Protocol<br>• Statistical Analysis Plan	Title Acronym:<br><br>Other Ids:<br>54792	Terminated	• Irritable Bowel Syndrome With Constipation	• Drug: SYN-010 21 mg<br>• Drug: SYN-010 42 mg<br>• Drug: Placebo	Study Type:<br>Interventional<br><br>Phase:<br>Phase 2<br><br>Study Design:<br>• Allocation: Randomized<br>• Intervention Model: Parallel Assignment<br>• Masking: Quadruple (Participant, Care Provider, Investigator, Outcomes Assessor)<br>• Primary Purpose: Treatment<br><br>Outcome Measures:<br>• Change From Baseline in the Weekly Average Number of Completely Spontaneous Bowel Movements (CSBM) Compared to the 12-week Treatment Period<br>• Proportion of Overall Responders During the 12-week Treatment Period<br>• Proportion of Overall Stool Frequency Responders During the 12-week Treatment Period<br>• Proportion of Overall Abdominal Pain Intensity Responders During the 12-week Treatment Period<br>• Proportion of Overall Bloating Responders During the 12-week Treatment Period<br>• Proportion of Patients Using Rescue Medication	Enrollment:<br>59<br><br>Age:<br>18 Years to 65 Years (Adult, Older Adult)<br><br>Sex:<br>All	• Cedars-Sinai Medical Center<br>• Synthetic Biologics Inc.	• Other<br>• Industry	Study Start:<br>December 24, 2018<br><br>Primary Completion:<br>October 9, 2020<br><br>Study Completion:<br>October 9, 2020<br><br>First Posted:<br>December 4, 2018<br><br>Results First Posted:<br>September 5, 2021<br><br>Last Update Posted:<br>July 13, 2022	• Cedars-Sinai Medical Center, Los Angeles, California, United States

	NCT Number	Title	Other Names	Status	Conditions	Interventions	Characteristics	Population	Sponsor/ Collaborators	Funder Type	Dates	Locations
27	NCT02857257	Transplantation of Anaerobic Cultured Human Intestinal Microbiota in Irritable Bowel Syndrome<br><br>Study Documents:	Title Acronym: ACHIM2<br><br>Other Ids: ACHIM2	Unknown status	• Irritable Bowel Syndrome	• Biological: ACHIM	Study Type: Interventional<br><br>Phase: Phase 2<br><br>Study Design:<br>• Allocation: N/A<br>• Intervention Model: Single Group Assignment<br>• Masking: None (Open Label)<br>• Primary Purpose: Treatment<br><br>Outcome Measures:<br>• Symptom relief according to irritable bowel syndrome-symptom severity scale (IBS-SSS)<br>• Differential bacterial species population as defined by 16S RNA<br>• Normalization of stool consistency as determined by the Bristol stool scale	Enrollment: 50<br><br>Age: 18 Years to 80 Years (Adult, Older Adult)<br><br>Sex: All	• Uppsala University<br>• Karolinska Institutet	• Other	Study Start: January 2015<br><br>Primary Completion: December 2018<br><br>Study Completion: December 2018<br><br>First Posted: August 5, 2016<br><br>Results First Posted: No Results Posted<br><br>Last Update Posted: August 8, 2018	• Mag-tarm/endoskopienheten Hötorget, Stockholm, Sweden
