# Supplementary material for: Irritable Bowel Syndrome and the Gut Microbiome: A Comprehensive Review
Source: J Clin Med. 2023 Mar 28;12(7):2558. doi: 10.3390/jcm12072558 (PMC10095554; doi:10.3390/jcm12072558)
Supplement: Supplementary file 1 [file jcm-12-02558-s001.zip › Supplemental File_Glossary IBS and Microbiome.pdf]

Supplemental File: Glossary

Functional Gastrointestinal Disorder (FGID): Persistent and recurrent gastrointestinal symptoms that exist in the absence of a known structural or biochemical abnormality of the gastrointestinal symptom. FGID can affect any portion of the GI tract including the esophagus, stomach and the intestines. Although not considered a psychiatric condition, psychiatric comorbidities including anxiety, depression, and PTSD are common.

Irritable Bowel Syndrome (IBS): A functional gastrointestinal disorder (FGID) characterized by chronic abdominal pain or discomfort and altered bowel habits. A diagnosis of IBS is clinically determined based on the Rome IV criteria, which defines IBS as recurrent abdominal pain that occurs on average of at least one day per week in the last three months involving two or more of the following: associated with defecation, change in stool frequency, or change in stool appearance.

Gut Microbiome: A group of microorganisms including viral, bacteria, fungi, and protozoa that exist in the digestive tract. These organisms are involved in maintaining gut homeostasis and immunomodulation of the GI tract. There is considerable variation of gut microbiome composition across humans which is believed to play a role in pathogenesis of gastrointestinal conditions.

Dysbiosis: Imbalance of the gut flora that can occur through the loss or overgrowth of a particular organism, reduction in microbial diversity, or gene mutations. Recent evidence suggests that gut dysbiosis may contribute to the pathogenesis of IBS

Biopsychosocial Model for Functional Gastrointestinal Disorders: A conceptual model which outlines how environmental, genetic, cultural, and psychosocial factors affect gastrointestinal physiology. Specifically, the interplay between these factors likely plays a role a role in the development of FGID including IBS by altering gut motility, sensation, immune and inflammatory response, gut microflora as well as diet.

Brain-Gut Axis: The bidirectional relationship between the nervous system and the gut that occurs via complex interactions between the endocrine system (hypothalamic pituitary adrenal axis), immune mediators (cytokines) and neurons allowing for direct communication between the brain and the gastrointestinal system. Dysregulation of the gut-brain axis has been linked to several gastrointestinal conditions including IBS.

FODMAP Diet: A diet low in FODMAPs – or fermentable oligosaccharides, disaccharides, monosaccharides, and polyols. FODMAPs are poorly absorbed in the small intestine, leading to increased water absorption and gas production via fermentation in the large colon which may contribute to symptoms of abdominal pain and bloating that patients with IBS experience. Adherence to a low FODMAP diet has been associated with a significant improvement in IBS symptoms.

Prebiotics: Prebiotics are indigestible compounds consisting of carbohydrates (fructooligosaccharides [FOS] and galactooligosaccharides [GOS]) which stimulate growth of bacteria. Prebiotics alter stool consistency, by increasing stool bulk and fecal water content which may be beneficial in constipation-predominant IBS.

Probiotics: Probiotics are live microorganisms that have beneficial properties specifically to the gut microbiota when consumed. Many studies have found significant improvement in IBS symptoms with use of probiotics; however, note significant heterogeneity as well as significant bias across studies, bringing into question their validity.

Fecal Microbiota Transplantation (FMT): The process in which stool from a healthy donor is transferred to the colon of a different individual with the intent of altering their microbiome. FMT has been explored as a potential treatment for IBS. It is not currently accepted as a first-line treatment for IBS given significant variation in efficacy across studies.
